# Performance of Xpert MTB/RIF and acid-fast bacilli smear microscopy for the diagnosis of pulmonary tuberculosis using bronchoalveolar lavage samples in negative or sputum-scarce adults in Colombia: a retrospective diagnostic accuracy study

**DOI:** 10.1186/s12879-025-10856-z

**Published:** 2025-04-09

**Authors:** Vanessa Sabella-Jiménez, Valentina L. Sabella-Jiménez, Valentina Restrepo-Espinosa, Juanita Eljadue-Flórez, Claredit Valentina Gallardo-Castro, Andrea Alexandra Silvera, Carlos Otero-Herrera, Hugo Andrés Macareno Arroyo, Jorge Acosta-Reyes, Jorge Luis Quintero Barrios

**Affiliations:** 1 Department of Public Health, Universidad del Norte, Barranquilla, 080001 Colombia; 2 Department of Medicine, Universidad del Norte, Barranquilla, Colombia; 3 Department of Internal Medicine, Universidad del Norte, Barranquilla, Colombia; 4 Department of Internal Medicine, Hospital Universidad del Norte, Barranquilla, Colombia; 5 Department of Pulmonology, Hospital Universidad del Norte, Barranquilla, Colombia

**Keywords:** Tuberculosis, Pulmonary tuberculosis, Lavage, Bronchoalveolar, Tests, Routine diagnostic

## Abstract

**Background:**

Limited literature exists on the diagnostic yield of traditional and molecular tuberculosis (TB) tests using bronchoalveolar lavage (BAL) specimens in high burden South American countries. The aim of the study is to determine the value of acid-fast bacilli (AFB) smear microscopy and Xpert MTB/RIF using BAL samples for diagnosing pulmonary TB (PTB) in negative or sputum-scarce adults.

**Methods:**

A cross-sectional, retrospective diagnostic accuracy study was carried out between January 1, 2015 and December 31, 2023. Demographic, clinical, imaging and microbiologic data were collected from consecutive medical records of initially negative or sputum-scarce adults, who underwent bronchoscopy due to presumptive PTB. Positive cultures were identified as *Mycobacterium tuberculosis*. Validity criteria was evaluated by sensitivity, specificity, positive (PPV) and negative predictive values (NPV), and Cohen’s kappa with their 95% confidence intervals using Wilson’s score method by OpenEpi Version 3.01.

**Results:**

Of the 701 medical records screened, 283 adults were included. Cough (89.59%) and fever (64.31%) were the most frequent symptoms. Smear microscopy obtained a sensitivity of 39.22% (95% CI 27.03–52.92), specificity of 98.26% (95% CI 95.61–99.32), PPV of 83.33% (95% CI 64.15–93.32), NPV of 87.94% (95% CI 83.39–91.37) and Cohen’s kappa of 0.472 (95% CI 0.3652–0.5788). Xpert MTB/RIF obtained a sensitivity of 91.67% (95% CI 80.45–96.71), specificity of 90.09% (95% CI 85.33–93.43), PPV of 67.69% (95% CI 55.61–77.8), NPV of 97.95% (95% CI 94.85–99.2) and Cohen’s kappa of 0.7191 (95% CI 0.5998–0.8384).

**Conclusions:**

Xpert MTB/RIF outperformed smear microscopy using BAL samples for the diagnosis of PTB in negative or sputum-scarce adults from a high burden Colombian setting. Due to rapid results, affordability and accessibility, particularly in resource-limited settings, smear microscopy should still be considered for BAL samples.

## Background

Tuberculosis (TB), a communicable disease caused by *Mycobacterium tuberculosis* (MTB) infection, is one of the leading causes of mortality worldwide [1]. Without timely diagnosis and proper treatment, mortality is high (~ 50%), thus highlighting the importance of early screening and diagnosis [1]. In 2021, 14,060 cases of TB were reported in Colombia, with a national incidence rate of 25.9 cases per 100,000 inhabitants [2]. A total of 51.5% of the territorial entities of departmental order of the country are of high burden for TB [3]. Likewise, human immunodeficiency virus (HIV) infection may lead to a higher risk and burden of TB, considering that TB and HIV coinfection is the second (12.1%) registered comorbidity of TB cases in Colombia [2].

In Colombia, confirmation of the diagnosis is obtained by demonstration of the mycobacteria through microbiologic studies from sputum samples, which can be produced spontaneously, through induction or gastric lavage [4]. However, sputum induction can fail to provide a specimen of adequate volume or quality in up to 20% of cases [5]. Hence, if TB is presumed but the results of microbiologic studies in sputum are negative and the quality or quantity of the sample is poor, obtaining bronchoalveolar lavage (BAL) samples through bronchoscopy is an alternative option [6] to improve the diagnostic accuracy and aid in the rapid diagnosis of pulmonary TB (PTB) patients [7].

Traditional TB diagnostic tools, such as culture and acid-fast bacilli (AFB) smear microscopy, have strengths and limitations. First, the *Mycobacterium* culture is generally considered the gold standard test for the diagnosis of TB [6]. However, TB culture methods are not widely available in high-burden settings [8], can take up to 4–8 weeks [6, 9], require a minimum of 10 bacilli per mL in order to identify bacterial colonies [10], and require extensive infrastructure, lacking in resource-limited settings [11]. Second, although AFB smear microscopy is a simple and fast procedure and has high specificity (approximately 98%) [12], the sensitivity can be low [13, 14], and a minimum of 10,000 bacilli per mL of sputum are required for detection [10].

Since 2010, the World Health Organization (WHO) recommends the Xpert MTB/RIF (Cepheid, Sunnyvale, CA, USA) for national TB programmes in developing countries, particularly for the diagnosis of PTB in HIV-infected individuals [15]. Even though it is an automated, disposable single-cartridge-based nucleic acid amplification test that is able to simultaneously detect MTB and susceptibility to rifampicin within 2–3 h [6], it is more expensive.

Few studies worldwide have evaluated the performance of Xpert MTB/RIF of BAL, particularly in individuals with sputum-smear negative TB [8]. This will be the first study in the colombian caribbean region, a high TB burden setting, to determine the value of AFB-smear microscopy and Xpert MTB/RIF tests using BAL samples for the diagnosis of PTB in sputum-smear negative or sputum-scarce patients, compared to the gold-standard culture test.

## Methods

### Study design

A phase III, cross-sectional, diagnostic accuracy study was carried out at Hospital Universidad del Norte, a tertiary hospital and high complexity center in the city of Soledad of Colombia’s caribbean region.

### Study setting

We retrospectively reviewed all consecutive medical records of adult patients over 18 years of age, who underwent bronchoscopy at Hospital Universidad del Norte between January 1, 2015 and December 31, 2023, and later included those with presumptive PTB, with initial negative sputum AFB smear microscopy and/or those sputum-scarce, and with collected BAL samples for microbiologic studies. Patients who previously received anti-tubercular treatment were excluded.

The population consisted of 3 subgroups: a) patients with complete clinical, imaging and laboratory data (AFB smear microscopy, Xpert MTB/RIF and culture results) from Hospital Universidad del Norte; b) patients with complete clinical and imaging data, who had the culture result available and either AFB smear microscopy (b1) or Xpert MTB/RIF (b2) results from Hospital Universidad del Norte; c) patients referred from other institutions to Hospital Universidad del Norte with presumptive PTB, whose medical insurance exclusively authorized the bronchoscopy procedure and the three microbiologic studies to be performed and reported at our center (after the procedure, patients were transported to their initial institution for continued care).

Presumptive PTB was considered if the patient presented with suggestive symptoms: persistent cough lasting more than 2 weeks, hemoptysis, unexplained weight loss, intermittent fever, night sweats, chest pain and/or shortness of breath. Furthermore, at least two chest computed tomography (CT) scan findings had to be described in the report to be considered presumptive of TB: tree-in-bud pattern or bronchial spread, cavitation, bronchiectasis, diffuse infiltrates, lymph node enlargement, hilar enlargement, atelectasis, lung nodule, pleural effusion, interstitial pattern, pulmonary consolidation and/or alveolar infiltrates.

### Procedures

Bronchoscopies were performed by a pulmonologist in an operating room under conscious sedation. The bronchoscope was placed into an airway of an affected lung segment for description of the observed respiratory anatomy. Lavage was performed with instillation of 0.9% sterile saline solution into a section of an affected lung segment. The returned aspirate was collected into a sterile tube for further processing. The BAL sample was then sent to microbiology for AFB smear microscopy, Xpert MTB/RIF, and either Lowenstein-Jensen or Ogawa-Kudoh methods for solid medium culture processing or the BACTEC™ MGIT 960™ (Mycobacteria Growth Indicator Tube) for liquid medium culture processing.

Samples of maximum 10 mL were processed according to standard laboratory protocols by decontamination with N-acetyl-L-cysteine and sodium hydroxide (NALC/NaOH) for 15 min and then neutralized by the addition of phosphate buffer. After centrifugation for 15–20 min at 3000 g, the supernatant was discarded and the pellet was suspended in 1 mL of phosphate buffer. With one drop, smear was performed, and 0.5 mL of the suspension was inoculated in an BACTEC™ MGIT™ tube system (Becton Dickinson, Sparks, Maryland, USA) [16] and 200 μL onto the surface of Löwenstein–Jensen (L–J) medium and incubated for 6 and 8 weeks, respectively. Some sediments were cultivated on 3% Ogawa solid medium for 8 weeks in 5–10% CO2 incubators. Weekly, each culture was read until positive growth of bacterial colonies (to assess presence of live bacilli) was observed [17], or until the 6–8 weeks were completed and no growth was observed. Positive cultures were identified as MTB and considered the gold standard for the diagnosis of PTB.

Likewise, direct smears from each BAL specimen were performed using Ziehl–Neelsen staining for AFB [18]. After applying the specimen to a clean glass slide and slowly spreading the liquid to make a thin film of 1 cm in diameter, it was dried, fixed at 80 °C for 15 min and saturated with carbolfuchsin stain. Next, the slide was heated, kept moist and steamed for 5 min. The film was washed with distilled water, decolorizing solvent and flooded with methylene blue counterstain for 20–30 s. After washing with distilled water and air drying the smear, the film was examined under oil immersion to observe red AFB [18].

The Xpert MTB/RIF assay was performed according to the manufacturer’s specifications (Cepheid, Sunnyvale, CA, USA) [19]. BAL fluid of 1 mL, without prior decontamination or centrifugation, was mixed with 2 mL of Xpert sample reagent, followed by incubation for 15 min at room temperature. Next, 2 mL of digested specimen was loaded into the Xpert MTB/RIF cartridge and test results were reported as ‘not detected’ or ‘detected’. It was considered positive if MTB was identified within 38 cycles [19, 20].

### Statistical analysis

Detailed demographic characteristics, HIV infection, and clinical and imaging characteristics were presented in number and percentages. Descriptive statistics were performed. Frequency analysis and centrality measures were performed for the age variable. Contingency 2 × 2 tables were conducted. Validity criteria -sensitivity, specificity, positive predictive value, negative predictive value, likelihood ratios and diagnostic accuracy- for the diagnosis of patients with presumptive PTB, as well as Cohen’s kappa, were calculated for AFB smear microscopy and Xpert MTB/RIF from BAL samples compared to the gold standard. Their 95% confidence intervals (CI) were estimated using Wilson’s score method by OpenEpi *(Open Source Epidemiologic Statistics for Public Health)*, Version 3.01, open source calculator [21].

## Results

**Fig. 1:**
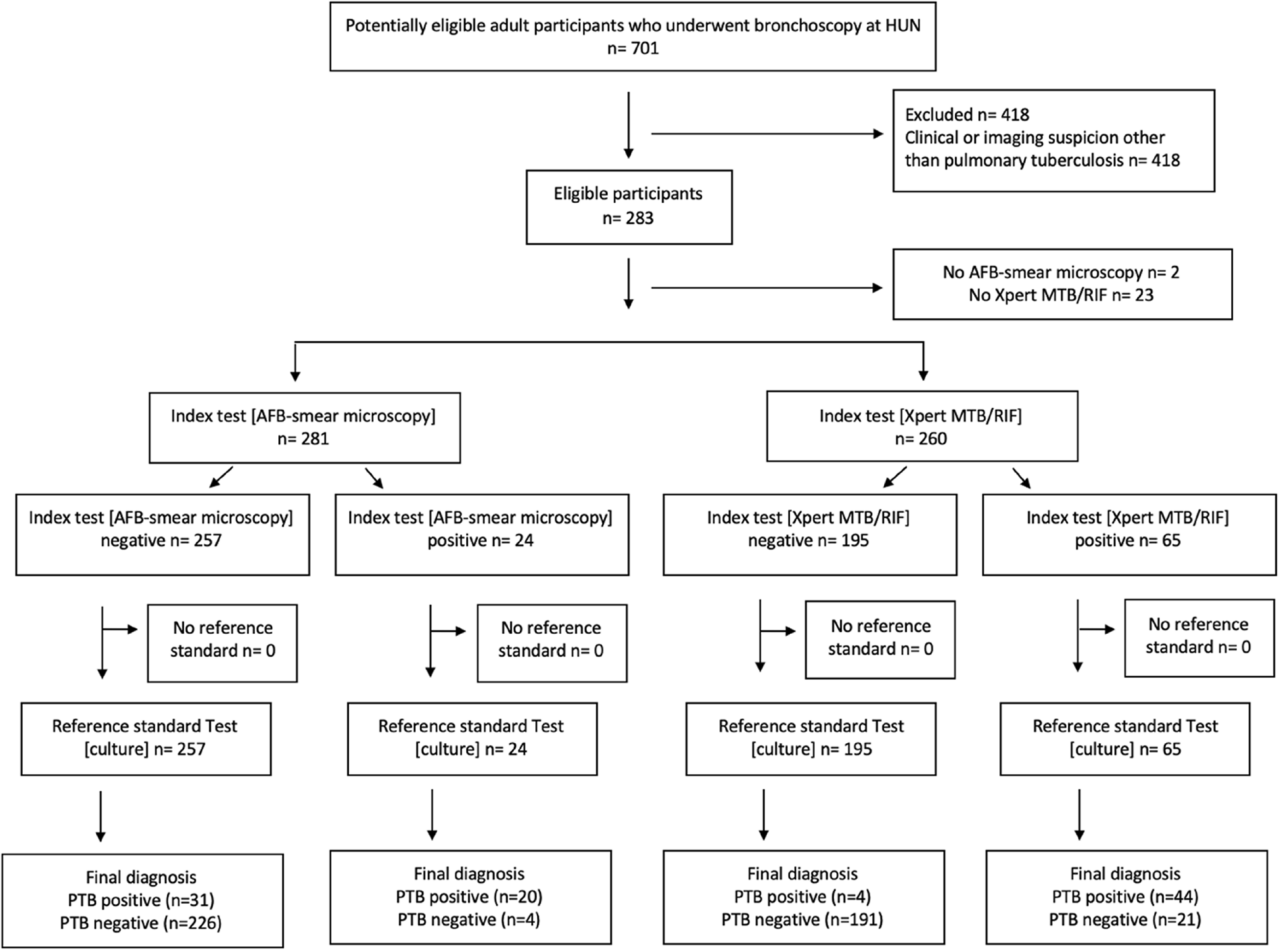
Standard for Reporting Diagnostic Accuracy (STARD) flow diagram of presumptive pulmonary tuberculosis cases included in the study. Abbreviation: HUN = Hospital Universidad del Norte, PTB = pulmonary tuberculosis, AFB = Acid-fast bacilli, MTB/RIF = Mycobacterium tuberculosis/Rifampicin

We screened 701 medical records of patients who underwent bronchoscopy, and included 283 medical records of patients who met the eligibility criteria. Of the 283 patients, the three subgroups were divided as follows: a) 244 patients with complete clinical, imaging and microbiological data; b) 23 patients with complete clinical and imaging findings, as well as culture and AFB smear microscopy results (b1), and 2 patients with complete clinical and imaging findings, as well as culture and Xpert MTB/RIF results (b2); c) 14 patients referred to our center for the bronchoscopy procedure and the three microbiology results (Fig. 1).

From the subgroup b, 23 Xpert MTB/RIF samples and 2 AFB smear samples were unavailable due to administrative issues with the patient’s medical insurance, which allowed samples to be taken by the specialist through the bronchoscopy procedure, but did not allow some of the test results to be processed or reported at our institution. Thus, they were referred to other medical institutions/laboratories for processing or report.

A total of 14 patients (subgroup c) with presumptive PTB were referred from other institutions to Hospital Universidad del Norte exclusively for bronchoscopies and processing of the three microbiological tests. Of these patients, clinical and imaging findings were not available at Hospital Universidad del Norte’s medical records since the patients’ information was kept at the initial institution. Even though patients were transferred back to their initial institution after the procedure for their continued care, the BAL culture, AFB smear microscopy and Xpert MTB/RIF were processed at our institution’s laboratory and thus results were available for review.

Overall, 50.53% of the 283 participants were male. The mean age was 55.65 years (SD ± 18.38) and 19.43% of patients had HIV infection (Table 1). Clinical and imaging characteristics for subgroups a, b1 and b2 are shown in Table 1.

**Table 1:** Demographic, clinical and imaging characteristics of subgroups

Demographic characteristics	*N* = 283^a^
**Mean age in years (SD)**	55.65 (18.38)
**Gender**	
Male (%)	143 (50.53%)
Female (%)	140 (49.46%)
**HIV** (%)	55 (19.43%)
**Clinical characteristics**	***N* = 269**^**b**^
**Symptoms** (%)	
Cough	241 (89.59%)
Fever	173 (64.31%)
Shortness of breath	153 (56.87%)
Weight loss	149 (55.39%)
Hemoptysis	58 (21.56%)
Chest pain	52 (19.33%)
Night sweats	45 (16.72%)
**Imaging characteristics**	***N* = 269**^**b**^
**Chest CT scan findings** (%)	
Tree-in-bud pattern	209 (77.69%)
Lymph node enlargement	130 (48.32%)
Pulmonary consolidation	112 (41.63%)
Bronchiectasis	111 (41.26%)
Lung nodule	103 (38.28%)
Atelectasis	94 (38.28%)
Cavitation	71 (26.39%)
Pleural effusion	54 (20.07%)
Alveolar Infiltrate	51 (18.95%)
Interstitial pattern	16 (5.94%)
Diffuse pulmonary infiltrate	13 (4.83%)
Hilar enlargement	8 (2.97%)

*Abbreviation*: *SD* Standard deviation, *HIV* Human immunodeficiency virus, *CT* Computed tomography

^a^Includes subgroups a, b1, b2 and c

^b^Includes subgroups a, b1 and b2

### Diagnostic accuracy of BAL AFB smear microscopy and Xpert MTB/RIF

Twenty culture-positive cases were identified as positive through AFB smear microscopy, while 44 culture-positive cases were identified as positive through Xpert MTB/RIF (Tables 2 and 3). Thus, resulting in a sensitivity of 39.22% (95% CI 27.03–52.92) for AFB smear microscopy, and 91.67% (95% CI 80.45–96.71) for Xpert MTB/RIF. The specificity of AFB smear microscopy was 98.26% (95% CI 95.61–99.32), while the specificity of the Xpert MTB/RIF assay was 90.09% (95% CI 85.33–93.43) (Table 4).

**Table 2:** Contingency table of the diagnostic performance of acid-fast bacilli smear microscopy of bronchoalveolar lavage samples for diagnosis of pulmonary tuberculosis

AFB smear microscopy	Positive culture	Negative culture	Total
Positive	20	4	24
Negative	31	226	257
Total	51	230	**281**

*Abbreviation*: *AFB* Acid-fast bacilli

**Table 3:** Contingency table of the diagnostic performance of Xpert MTB/RIF of bronchoalveolar lavage samples for diagnosis of pulmonary tuberculosis

Xpert MTB/RIF	Positive culture	Negative culture	Total
Positive	44	21	65
Negative	4	191	195
Total	48	212	**260**

*Abbreviation*: *MTB/RIF* Mycobacterium tuberculosis/Rifampicin

**Table 4:** Diagnostic accuracy of Xpert MTB/RIF assay and AFB smear microscopy using bronchoalveolar lavage samples with culture as gold standard for the diagnosis of pulmonary TB

	**Test**	
	**AFB smear microscopy**	**Xpert MTB/RIF assay**
**Sensitivity**	39.22% (27.03–52.92)	91.67% (80.45–96.71)
**Specificity**	98.26% (95.61–99.32)	90.09% (85.33–93.43)
**Positive predictive value**	83.33% (64.15–93.32)	67.69% (55.61–77.8)
**Negative predictive value**	87.94% (83.39–91.37)	97.95% (94.85–99.2)
**Diagnostic accuracy**	87.54% (83.17–90.91)	90.38% (86.19–93.4)
**Positive likelihood ratio**	22.55 (11.87–42.85)	9.254 (8.395–10.2)
**Negative likelihood ratio**	0.6186 (0.5806–0.6591)	0.0925 (0.0566–0.1512)
**Cohen’s kappa**	0.472 (0.3652–0.5788)	0.7191 (0.5998–0.8384)
**TB prevalence**	18.14%	18.46%

*Abbreviation*: *AFB* Acid-fast bacilli, *MTB/RIF* Mycobacterium tuberculosis/Rifampicin, *TB* Tuberculosis

While AFB smear microscopy had a positive predictive value (PPV) of 83.33% (95% CI 64.15–93.32) and a negative predictive value (NPV) of 87.94% (95% CI 83.39–91.37), Xpert MTB/RIF had a PPV and NPV of 67.69% (95% CI 55.61–77.8) and 97.95% (95% CI 94.85–99.2), respectively. As previously stated, the number of patients who had AFB smear microscopy results available was different from the number of patients who had Xpert MTB/RIF results available. Despite this, the prevalence of PTB for both test populations were similar (18.14% and 18.46%), respectively (Table 4).

The diagnostic accuracy of AFB smear microscopy and Xpert MTB/RIF, relating to the ability of the tests to correctly identify pulmonary tuberculosis, was similar, with values of 87.54% (95% CI 83.17–90.91) and 90.38% (95% CI 86.19–93.4), respectively (Table 4). It was 22.55 (95% CI 11.87–42.85) times more likely to obtain a positive AFB smear microscopy result in patients with PTB than in patients without PTB (Table 4). On the contrary, it was 10.81 times less likely to obtain a negative Xpert MTB/RIF result in patients with PTB than in patients without PTB. Finally, Cohen’s kappa reported a moderate reliability for AFB smear microscopy with a result of 0.472 (95% CI 0.3652–0.5788), while Xpert MTB/RIF assay’s result reported a substantial reliability with a result of 0.7191 (95% CI 0.5998–0.8384).

### Detection of isoniazid and rifampicin resistance

Two patients had isoniazid mono-resistance and none had rifampicin resistance. Those two patients were culture positive, received treatment as per international guidelines, and were closely followed-up by infectious diseases, pulmonology and internal medicine providers.

## Discussion

Our findings support the use of molecular testing as well as the implementation of traditional diagnostic tools, such as AFB smear, in BAL specimens in the scenario of sputum-scarce or sputum-negative adults for the improvement of TB diagnosis. Considering that the diagnosis of PTB may be limited by patients’ failure to produce sputum (spontaneously or induced) [22], difficulty in obtaining sufficient sample for analysis [22], the low sensitivity of sputum AFB-smear microscopy [23], the incapacity of smear microscopy to differentiate between MTB and nontuberculous mycobacteria [22], the culture’s time-consuming procedure [6], and medical institutions lacking rapid molecular testing, the collection of BAL specimens are a valid alternative in the context of presumptive PTB and a negative sputum.

While we considered a PTB diagnosis if the individual had a positive solid or liquid culture test, in a recent systematic review that analyzed the performance of Xpert MTB/RIF using BAL fluid, the majority of studies used liquid culture as the reference standard [6]. Only one retrospective study performed in Korea in 2018 used both cultures as reference standards [24]. Evidence from South African [8, 25], Indian [26, 27], Pakistani [28] and Egyptian [29] adults with sputum negative smear results, compared to only liquid culture, are also among the limited worldwide data regarding the diagnostic accuracy of TB diagnostic tests using BAL samples.

Furthermore, literature on the diagnostic yield of TB tests of BAL specimens among high TB burden South American territories, such as Colombia, is also limited. In South America, two diagnostic retrospective studies of positive-smear adults were conducted in Brazil, with solid or liquid culture as their gold standard references [30, 31]. A more recent study in Brazil evaluated sputum smear-negative patients, or those with insufficient sputum, who underwent BAL to obtain Xpert MTB/RIF, AFB staining and solid culture results [22]. Even though a retrospective study was conducted in Medellín, Colombia and evaluated the diagnostic yield of polymerase chain reaction, compared to solid and liquid culture on BAL samples of presumptive PTB patients, it failed to provide information on whether samples were obtained from initially sputum-smear negative or sputum-scarce patients [10].

Concerning TB test results, AFB smear microscopy on BAL was highly specific, yet poorly sensitive. Thus, the utility of this test is based upon a positive result, and with a high specificity, PTB can be confirmed rapidly in the setting of sputum-scarce or sputum smear-negative adults. In South African [8] and Brazilian [8, 22] studies, sensitivities for AFB smear microscopy were higher (57.7% and 68.1%, respectively) and overall specificities were similar to ours (99.3% and 96.1%, respectively). While in India, sensitivity was considerably lower (18.8%) [26], compared to our percentage. Moreover, other values of the diagnostic test also vary globally. In a Brazilian study, a slightly higher PPV (88.2%) and similar NPV result (87.5%) [22] were observed when compared to solid culture. In contrast, in India, compared to liquid culture, PPV and NPV were considerably lower (50% and 74%, respectively) [26] than ours. The prevalence of culture-positive PTB in adults with AFB smear results (18.14%) was lower than observed in India (26.6%) [26], yet higher than Brazil (15.7%) [22].

Conversely, in the present study the Xpert MTB/RIF assay provided high sensitivity, specificity and overall accuracy, considering that HIV-infected patients were also included. The higher sensitivity of Xpert MTB/RIF, compared to AFB smear microscopy, exhibits an advantage in excluding PTB. Other advantages offered by the Xpert MTB/RIF assay, such as rapid results that allow faster treatment initiation and the rifampin-resistance detection, undermine the higher costs of equipment and their maintenance, compared to AFB smear microscopy.[22] Studies in Brazil [22], Pakistan [28] and South Africa [8] have observed a higher sensitivity (81.82%, 91.86%, 92.6%, respectively) of the Xpert MTB/RIF assay than that of the smear microscopy in BAL samples, despite overall similar or lower specificity. Furthermore, the Xpert MTB/RIF Ultra assay (Cepheid, Sunnyvale, CA, USA), the latest Xpert technology, was developed using two different multicopy amplification targets (IS6110 and IS1081) and a larger reaction chamber, which also aims to improve sensitivity for MTB detection [32, 33]. Xpert MTB/RIF Ultra using bronchial washing fluid, has been implemented in detecting smear-negative PTB, showing higher sensitivity compared to culture [32]. This underscores its potential as another valuable tool for early PTB detection in the setting of smear-negative PTB, with the hope that more countries in South America will adopt its use to improve TB diagnosis and treatment.

The laboratory at Hospital Universidad del Norte adheres to the required quality control standards and participates in regular internal and external quality assessments to ensure the reliability and accuracy of its procedures. However, despite these rigorous standards, discrepancies can still occur between diagnostic methods due to various factors. In the present study, discrepancies were observed between AFB smear microscopy, Xpert MTB/RIF and culture methods. In 4 cases in our study, AFB smear microscopy yielded positive results, as well as Xpert MTB/RIF, while culture results were negative. Two of these samples, cultured on solid media, had a smear positivity level of 1 +, indicating a lower bacterial load, which may explain why bacteria were detected by microscopy but not by culture. In other cases, AFB smear microscopy can identify MTB without a positive culture result, particularly in HIV-infected individuals, where immunosuppression can favor coinfection with other pathogens, such as non-tuberculous mycobacteria [22]. In our study, and these particular cases, non-tuberculous mycobacteria were not tested. Likewise, positive smears, even with presumptive imaging, have also been reported with false-negative Xpert MTB/RIF results, suggesting that Xpert-negative does not perfectly rule out the noninfectious nature of presumptive PTB patients [34].

Additionally, for the Xpert MTB/RIF assay, approximately one-third of patients with a positive result had a negative solid or liquid culture. Although culture methods are considered the gold standard, they can occasionally fail to detect cases, particularly when bacterial load is low, there are higher threshold values (low concentrations of MTB DNA) [22], or when anti-tubercular therapy or other antibiotics were previously administered [26]. In some cases, MTB/RIF positive and culture negative results are further assessed with Genotype MTBDR*plus* assay, which detect MTB complex DNA [8]. Inherent differences in diagnostic testing measurements can also be potential sources of discordance between methods. In this case, Xpert MTB/RIF is highly sensitive and can detect *M. tuberculosis* DNA even when culture methods do not.

In our study, 19.43% of the participants with TB had HIV coinfection, which is higher compared to the stable 12.06% proportion indicator of TB-HIV coinfection in Colombia [35]. However, in a hospital setting, a greater number of individuals with HIV-related events can be observed compared to the general population. Poor TB treatment outcomes in high-burden countries, including high mortality rates [36], also exhibit the importance of early TB detection and the subsequent need for treatment in people living with HIV.

The frequency of clinical findings can vary to some extent, potentially reflecting regional disparities in PTB epidemiology. In the present study cough was the most frequent symptom (89.59%), followed by fever (64.31%), which aligned closely with prior research conducted in Medellín [10], India [27] and in Egypt [29]. Therefore, clinicians should be particularly vigilant for classic symptoms, such as cough and fever, when considering PTB in the setting of sputum smear-negative or sputum scarce adults. Nonetheless, local epidemiological data that may influence symptom presentation should also be evaluated. Disparities in symptom frequencies among various territories suggest differences in clinical manifestations and emphasize the complexity of TB epidemiology. For example, shortness of breath was observed at a lower frequency in a study in India (27%) [27], compared to ours (56.87%). Similarly, weight loss, present in 55.39% of our study participants, has also exhibited varying frequencies in different regions, by being present in 59.3% of cases in a study in Brazil [22] and in 78% of cases in a study in Egypt [29].

Regarding imaging findings, we only described CT scan findings to identify radiological signs compatible with presumptive PTB, similar to studies in Korea [24] and China [37], since CT is more sensitive than chest radiography in the detection and characterization of subtle localized or disseminated parenchymal disease and mediastinal lymphadenopathy [38]. Tree-in-bud pattern, lymph node enlargement, pulmonary consolidation and bronchiectasis were the most frequent findings, which differ from the studies of Korea [24] and China [37], in which the most common findings were cavitation and pulmonary sequestration. Even though cavitary pulmonary disease is classically associated with MTB [39], global frequencies may vary, with cavitation present in 26.39% of the cases in the current study, in 60% of cases in a study in Egypt [29] and only in 17.9% in a study in Brazil [22]. Similar to results in Egypt [29] and South Africa [25], consolidation was among the most common findings. In contrast, lymph node enlargement or lymphadenopathy was found in South Africa [25], India [26], and Egypt [29] to a lesser degree, compared to our frequency.

Our study has some limitations. First, it was a single-center study, which limits the generalizability of the data. Second, because it was a retrospective study, clinical data was limited to that available in the medical records. Third, 25 cases had only two results available from the methods evaluated. Therefore, missing results on either the AFB smear test or the Xpert MTB/RIF assay created two population sizes and TB prevalences for the evaluation of the diagnostic performance of each test. Fourth, 14 adults had no clinical or imaging results. Among the strengths, the study was performed at a tertiary, high complexity, teaching hospital in a high-TB-burden area, where tools are available for the investigation and accurate diagnosis of cases of TB. In addition, culture results were available for all consecutive cases, compared to at least another test from the BAL sample. Finally, regardless of unavailable clinical and imaging characteristics, all adults who were referred from other institutions with presumptive PTB were included in the diagnostic accuracy section of the study.

## Conclusions

The Xpert MTB/RIF assay (90.38% CI 86.19–93.4) outperformed AFB smear microscopy (87.54% CI 83.17–90.91) in BAL samples in our study. However, AFB-smear microscopy, should still be performed in BAL samples in smear-negative or sputum-scarce patients, considering its rapid results, affordability, accessibility and availability, particularly in resource-limited settings. Furthermore, local and regional epidemiology should be considered when presuming PTB, in order to provide accurate diagnoses and prompt treatment.

## Data Availability

The data used and analyzed in this study is available by reasonable request to the corresponding author, subject to institutional permission.

## Abbreviations

TB: Tuberculosis
BAL: Bronchoalveolar lavage
AFB: Acid-fast bacilli
PTB: Pulmonary tuberculosis
PPV: Positive predictive value
NPV: Negative predictive value
MTB: Mycobacterium tuberculosis
HIV: Human immunodeficiency virus
WHO: World Health Organization
CT: Computed tomography
CI: Confidence intervals
